# Fast single-cell biochemistry: theory, open source microscopy and applications

**DOI:** 10.1088/2050-6120/ab3bd2

**Published:** 2019-08-29

**Authors:** Andrew L Trinh, Suzan Ber, Annie Howitt, Pablo Oriol Valls, Maximilian W Fries, Ashok R Venkitaraman, Alessandro Esposito

**Affiliations:** MRC Cancer Unit, University of Cambridge, Cambridge, United Kingdom

**Keywords:** fast TCSPC, TDC, open-microscopy, FLIM, biochemistry

## Abstract

Fluorescence lifetime sensing enables researchers to probe the physicochemical environment of a fluorophore providing a window through which we can observe the complex molecular make-up of the cell. Fluorescence lifetime imaging microscopy (FLIM) quantifies and maps cell biochemistry, a complex ensemble of dynamic processes. Unfortunately, typical high-resolution FLIM systems exhibit rather limited acquisition speeds, often insufficient to capture the time evolution of biochemical processes in living cells. Here, we describe the theoretical background that justifies the developments of high-speed single photon counting systems. We show that systems with low dead-times not only result in faster acquisition throughputs but also improved dynamic range and spatial resolution. We also share the implementation of hardware and software as an open platform, show applications of fast FLIM biochemical imaging on living cells and discuss strategies to balance precision and accuracy in FLIM. The recent innovations and commercialisation of fast time-domain FLIM systems are likely to popularise FLIM within the biomedical community, to impact biomedical research positively and to foster the adoption of other FLIM techniques as well. While supporting and indeed pursuing these developments, with this work we also aim to warn the community about the possible shortcomings of fast single photon counting techniques and to highlight strategies to acquire data of high quality.

## 1 Introduction

**Figure 1:**
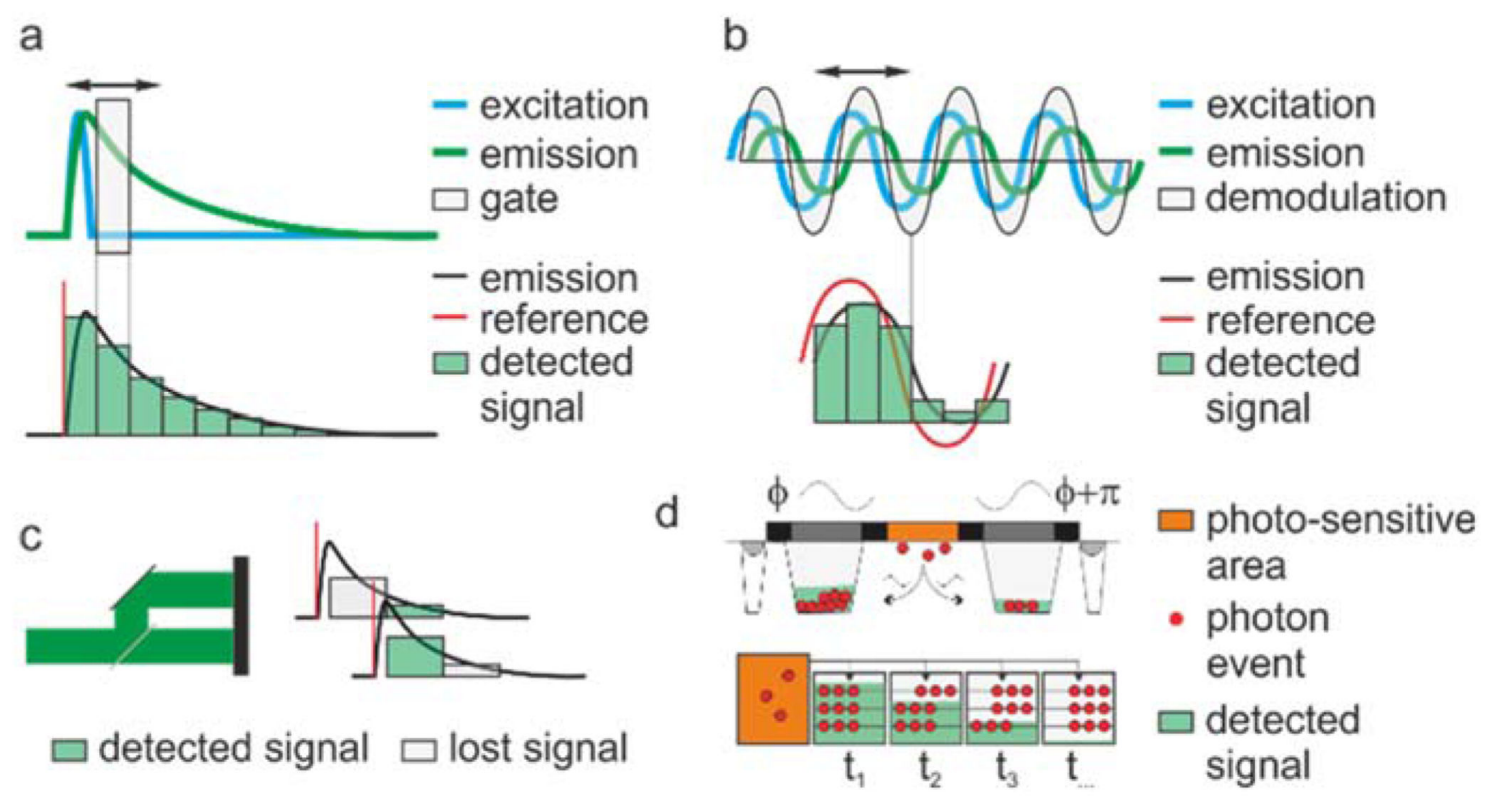
FLIM technologies and acquisition speed. Fluorescence lifetime sensing can be achieved in the time- (a) or frequency- (b) domain. The speed of FLIM is limited by scanning, either the physical scanning of an image or the scanning of the temporal properties of fluorescence emission. The speed of a wide-field microscope in FLIM is often limited by the need of scanning time-gates or phase-delays to reconstruct the emitted signal in comparison to a reference signal. In these schemes, light is lost at every (gated) exposure. However, scanning can be avoided by gating simultaneously in different time-windows split-images of the same objects (c), improving speed albeit retaining signal losses. More recent technologies permit the signal to be properly histogrammed in-pixel both for frequency- (top) and time- (bottom) domain application, providing the speed of wide-field microscopes and minimising losses (d).

Fluorescence lifetime imaging microscopy (FLIM) permits resesrchers mapping cell biochemistry in living cells, for instance, by detecting the state of naturally fluorescent (often meatbolic related) molecules sch as NADH, or by measuring the concentration of analytes, protein-protein interactions, enzymatic activities, conformational changes of molecules, pH and viscosity with the use of fluorescent probes [1]. Historically, technical limitations in FLIM have neccessitated the compromise between high spatial resolution, high temporal resolution, and the precision of lifetime measurements. Typical wide-field FLIM microscopes required only a few seconds of exposure to excitation light per image, during which, intensified gated or modulated cameras temporally slice-through exponentially decaying (time-domain detection [2], figure 1(a)) or sinusoidally modulated fluorescence signals (frequency-domain detection [3], figure 1(b)). Highly specialised prototypes provided acquisition speeds in excess of video-rate by utilising image-splitters and a single camera capable of acquiring a time-stack in a single shot [4–7] (figure 1(c)). However, even when operated in such single-shot modality, intensified cameras lose a significant amount of light by gating photons off during acquisition (figures 1(a), (c)). Often, these losses results in lower precision than techniques implemented with laser scanning microscopes. Laser scanning microscopes can provide higher resolution than conventional wide-field microscopes with the additional advantage that electronics and optical arrangements for single-pixel detection can grow in complexity compared to two-dimensional imagers (e.g., fast and precise digitizers and hyperspectral detection).

The gold-standard for FLIM in laser scanning microscopy is time-correlated single-photon counting (TCSPC) where each photon is counted and timed relative to the excitation pulses. This iterative process results in the reconstruction of the probability density function (*pdf*) of a fluorescence decay from which decay constants can be estimated. TCSPC has provided high precision, accuracy and resolution for FLIM; however, the acquisition speed of TCSPC has been comparatively slow until recently because of the long dead-times (*i.e.*, the time after the detection of one photon during which a system is insensitive to the detection of a second photon) of detectors and electronics, and the requirement of detecting no more than one photon-event per excitation pulse. Here, we will collectively refer to these types of saturation with the term ‘pulse pile-up’ [8]. Early systems exhibited microsecond long dead-times [9] but since the last few decades, the dead-time of a typical PMT or a multi-channel plate PMT used for TCSPC is about 10 ns and 200 ns, respectively, while the dead-time of the counting electronics is about 100 ns [10]. With the commercial adoption of hybrid PMTs in laser scanning microscopy, the bottle-neck in laser scanning FLIM acquisition rates remained the dead-time of the electronics, since hybrid PMTs have virtually no dead-time and can be operated at several tens of megahertz. To address this limitation, several strategies can be used to decrease the dead-time of the electronics. For instance, time-gating, which is commonly used in wide-field systems, has also been used in single photon counting laser scanning applications [10, 11]. Time-gating can be considered a special case of direct-to-histogram TCSPC where the timing information of individual photons is not measured but is instead directly histogrammed, simplifying the electronics and reducing the electronic dead-time [12, 13]. Electronic dead-times have also been improved through the use of field-programmable gate arrays implementing digital frequency domain FLIM [14]. Time-to-digital converters (TDCs) are a cost-effective off-the-shelf solution to reducing the electronic dead-time in laser scanning FLIM. Futhermore, TDCs with the capability to time multiple photons events per excitation pulse permits TCSPC system to overcome the tratisional limitaion of detecting no more than one photon per excitation cycle. These ‘multi-hit’ TDCs are akin to integrated timers triggered by a laser pulse and can record multiple stop events per pulse in response to the detection of individual photons. The minimal pulse-to-pulse time that can be resolved (pulse-pair resolution) is as low as 5 ns for commercially available and cost-effective devices, making fast TCSPC accessible.

Although we provided a brief and certainly incomplete overview of more established FLIM detection systems, the ecosystem of time-resolved microscopy is much more complex and ever-growing. Arrayed SPAD detectors capable of performing time-gating or TCSPC in-pixel [15–17] or on-chip [18, 19] speed-up acquisition times in FLIM while maintaining the precision of these techniques are used for wide-field imaging (figure 1(d)). Some of these technologies have reached commercial maturity, with time-of-flight ranging technologies for frequency- [20–24] (e.g., the PCO.FLIM by PCO GmbH and the Toggel by Lambert Instruments BV) and time- [25, 26] (e.g., the SPC2 camera by Micro Photon Devices and the PF32 camera by Photon Force) domain detection now available to implement video-rate wide-field FLIM. Also, the use of fast TDCs and ultra-fast digitizers have led to recently availabe fast laser scanning FLIM implementations, the RapidFLIM by PicoQuant GmbH and the FALCON by Leica Instruments GmbH, respectively. Both systems can deliver fluorescence lifetime sensing in seconds rather than minutes, and other companies, e.g. Becker and Hickl are providing means to reach high count-rates by reducing the overall dead-time of a system through multiplexing several high-end TCSPC electronics that individually still retain a high dead-time.

The need for high count-rates in FLIM with laser scanning microscopes is often disputed. Therefore, we will first introduce a basic theoretical description of the several advantages that can be achieved with new fast electronics. While recently developed commercial systems will likely further popularize FLIM, the costs of such high-end systems might limit their adoption. Therefore, we will then describe a custom multi-hit TDC-based TCSPC (nicknamed ELIS) capable of imaging four colours at high precision and high speeds [27]. This open platform has been in use for several years in our laboratory and might serve as a guide for others wanting to upgrade a laser scanning microscope (commercial or prototyped on an open-platform such as reported in [28]) to high-resolution, high-speed TCSPC-based FLIM using off-the-shelf and cost-effective components.

## 2 Methods

### 2.1 Microscopy

Details on the fast TCSPC system are described in section 4. All images presented in this work were acquired at a 256 × 256 pixel resolution at a speed of 400 Hz/line and a frame acquisition time of ~0.6 s. Measurements were done with a 40x oil immersion objective (Leica HCX PL APO CS NA = 1.25) at room temperature or, for live cell imaging, with a 100× oil immersion objective (Leica HCX PL APO CS NA = 1.4) at 37 °C, with two-photon excitation. For both objectives, Type 37 immersion oil by Cargille Laboratories (#16237, McCrone, UK) was used. The IRF of the system was measured by imaging crystals of potassium di-hydrogen phosphate (KDP, #P/4800/53 Fisher Scientific). A 200 *μ*l volume of a KDP supersaturate acqueos solution was placed at the centre of an open glass-bottomed chamber (#P35GC-1.5–14-C, MatTek) and allowed to evaporate. The KDP crystals were excited at 840 nm and imaged through two short-pass filters (SP680 by Leica and FES0450–1 by Thorlabs).

Count-rate estimates were determined using a fluorescent acrylic plastic slide (blue, Chroma Technologies), using the Leica CFP/YFP filter cube (SP680, band pass 483/32, dichroic BS505 and band pass 535/30) and excited at 840 nm. The detected photon-counts were divided by the effective pixel dwell time of the microscope. To estimate this value, we first multiplied the nominal frame time to the duty cycle of the imaging (65%) as estimated by the positive duty cycle of the blanking trigger utilised my the confocal microscope to switch-off excitation power during the retracing of the scanners (not shown). As a Leica SP5 confocal microscope exhibits an inhomogeneous pixel dwell time across the image, we further normalised each pixel intensity by the relative pixel dwell time determined imaging the fluorescent plastic at very low excitation rates to avoid saturation (not shown). Live cell imaging was peformed at 37 °C, with excitation at 840 nm and using the Leica CFP/YFP filter set already described.

### 2.2 Monte Carlo simulations and numerical methods

We ran Monte Carlo simulations to model the effects of dead-time and multi-hit capabilities at very high count rates in MATLAB (MathWorks). The code (‘*MAF*_*MC*_*sims.m*’) is available at the GitHub repository ‘alesposito/ELIS’. The photon arrival timing was simulated using a probability distribution function (*pdf*) for a single exponential decay as defined by Kollner and Wolfrum [29]:

(1) $$\text{pdf}\left(t_{i}\right)=P_{N}e^{-\frac{iT}{\tau k}}\frac{e^{-\frac{T}{\tau k}}-1}{1-e^{-\frac{T}{\tau }}}$$

where *t_i_* is the histogrammed time with bin number *i*, *k* is the maximum number of bins, *T* is the period of the laser and τ is the fluorescence lifetime. We amended the original definition with the factor *P_N_*, *i.e.* the probability to detect one photon within the period *T*. To obtain a realistic *pdf*, this decay was convolved with a Gaussian instrument response function centred around 1 ns and with a standard deviation of 0.1 ns (~230 ps FWHM). For all simulated conditions, we set k = 128, T = 12.5 ns and τ = 3 ns. Changes to these parameters do not alter the conclusions we report. Here, we performed a simple parameter sweep, with *P_N_* set at 0.01, 0.1 and 1 to simulate count-rates that are considered safe with TCSPC (0.01) or values that are typically avoided because of the significant losses of photon counts (e.g., precision) and accuracy. We also changed *t_d_* from 100 ns to 2.5 ns, to simulate ‘slow’ *versus* ‘fast’ electronics and the capability to count multiple photons per laser pulse, as just a single-hit or 32-hits spanning ten sequential laser periods (10 times T). A train of one million laser pulses was then simulated, resulting in 12.5 ms long Monte Carlo simulations at a resolution of ~100 ps (i.e., T/k). At each step, a random number generator was used to draw a number (*n_rnd_*) from a uniform distribution within the (0, 1) interval. When pdf(mod(t_i_, T)) ⩾ n_rnd_, a photon-event was triggered. The delay between this event and the simulated laser pulse was computed using a modulo operation with the laser period T as the time base for the decays. Photon arrival times were then histogrammed to generate a reference trace, *i.e.* the decay that should be ideally measured in the absence of pulse pile-up. To build a trace accounting for the dead-time of the electronics, when a photon was detected, the histogramming of photons in the ‘real’ trace was suspended for a period equal to *t_d_*. Furthermore, when a photon was detected, no other photon was counted within the remainder of a laser period (single-hit operation). However, when simulating multi-hit capabilities, this latter constraint was removed thus allowing for a maximum of 32 photon-events to be counted and histogrammed over 10 consecutive laser pulses.

The numerical evaluation of equation (4) was also performed with MATLAB with code (‘*MAF*_*A*_*numerical.m*’) available at the GitHub repository ‘alesposito/ELIS’.

### 2.3 Live cell imaging

#### 2.3.1 Cell culture

HeLa cells (CCL 2, European Collection of Cell Cultures #93021013; STR profiled through the Cambridge Institute CRUK services) were cultured in DMEM (Gibco) supplemented with 10% FBS and 1% Penicillin and Streptomycin, at 37° and 5% CO_2_. Cells were passaged every 3–4 days.

#### 2.3.2 Transfection and imaging of mitATeam1.03

HeLa cells were plated in 2-well LabTek II chambered coverglass slides (Nunc) at a density of 100,000 cells per well. After 24 h, cells were transfected with a mitochondrially targeted ATP sensor (mitAteam 1.03 [30]) by JetPRIME (Polyplus) following the manufacturer’s protocol. Briefly, for each well, 1.6 *μ*g of DNA was mixed with 150 *μ*l JetPRIME buffer and 3.2 *μ*l JetPRIME reagent and incubated for 10 min, then added drop-wise onto cells with wells containing 2 ml of media. After 4 h, cells were washed and supplemented with fresh growth medium to minimize toxicity. Cells were imaged 24 h later, in imaging media consisting of Leibovitz L-15 media (Gibco) supplemented with 10% FBS and 25 mM Glucose (Sigma). MitAteam was generated by inserting two copies of the cytochrome c oxidase subunit VIII mitochondrial targeting sequence in tandem (synthesized with the GeneArt String DNA fragment service by ThermoFisher) between the HindIII and BamHI restriction sites of the original ATeam plamids, a gift from Takeharu Nagai (AddGene, ##51958 [31]). Binding of ATP induces a conformational change that result in differences in FRET efficiencies.

#### 2.3.3 Transfection and imaging of Aurora Kinase B sensor

HeLa cells were seeded 80,000 per well in Ibidi 2-wells chambered slides. The next day cells were transfected with centromere-targeted (CENP-B fusion, a kind gift from Prof. Michael Lampson at the University of Pennsylvenia) Aurora Kinase B sensor [32] using JetPRIME reagent (Polyplus), according to manufacture’s guidelines. Briefly, for each well, 200 *μ*l jetprime buffer was mixed with 2 *μ*g of DNA and 6 *μ*l of JetPRIME reagent and incubated for 10 min Subsequently, 2 ml of growth media were added to the mix and used to replace cell media in the Ibidi chamber. After 4 h, cells were washed and supplemented with fresh growth media to minimize toxicity. 24 h later cells were treated with 50 nM of Poloppin I, an allosteric inhibitor for Polo-like Kinase-1 [33], for 16 h in imaging media. Imaging was performed at the end of 16 h incubation. The Aurora Kinase B sensor is a typical kinase sensor, the fusion of a donor-acceptor pair (CyPet and YPet in this case) with a linker containing one epitope that can be phosphorylated by Aurora B and a phosphobinding domain. Phosphorylation of the substrate cause a conformational changes that result in different FRET efficiencies.

### 2.4 Image analysis

Image analysis was carried out with MATLAB using custom open-source code—the ELIS toolbox—described in detail in section 4. The figures presented in this work were prepared with *ad hoc* code utilizing simple projections of the multidimensional data stacks summing over different dimensions. Fluorescence lifetime values were calculated using the phasor approach [34, 35]. Briefly, this method applies a Fourier transform to the intensity decay trace of each pixel. The real and imaginary parts of the transform are represented on a two-dimensional plot, which can be used to compute the phase and modulation of the signal. From there, lifetime values were determined from the phase and modulation. In this work, the phase lifetimes were represented. FLIM images shown in figure 7 were computed after a convolution averaging filter with kernel of 2 × 2, 2 × 2 and 3 × 3 and a threshold of minimum photon counts equal to 800, 200 and 80 photons for the high count-rate (41 s accumulation), high count-rate (6.4 s accumulation) and low count-rate (41 s accumulation). FLIM images in figure 8 were computed after a convolution averaging kernel of 5 × 5 and a threshold of minimum photon counts equal to 1,800.

## 3 Theory of fast acquisition FLIM

### 3.1 Acquisition throughput

For a general description of the theory of FLIM, we refer to other papers [1, 36, 37]. Here, we aim to provide theoretical considerations on precision, accuracy and acquisition speed of FLIM systems, particularly to justify our engineering choices. We and others have described the Fisher information content in FLIM and the highest attainable precision as inferred by the Cramer-Rao bound [10, 29, 38–40]. The performance of a FLIM system can be conveniently described with the figure-of-merit *F* [10], *i.e.* the ratio between the relative error in the determination of the fluorescence lifetime (*σ_τ_ τ*^−1^) and the smallest possible relative noise that can be achieved considering shot-noise in single-photon counting (*N*^−1/2^) when *N* photons are counted:

(2) $$F=\sigma_{\tau }\tau^{-1}\sqrt{N}$$

*F* converges to one for a system that uses information efficiently, while for larger values of *F*, a system is less efficient and requires more photons to attain a given signal-to-noise ratio (SNR). Setting a target SNR level (*SNR_0_*), the number of photons required to attain SNR_0_ is $N=F^{2}SNR_{0}^{2}.$ The exposure time (or cumulative pixel-dwell time) required to achieve this photon-count is $T_{0}=F^{2}SNR_{0}^{2}k_{0}^{-1},$ where *k_0_* is the average count-rate in the absence of pulse pile-up. However, significant photon-losses might occur in the presence of the pulse pile-up caused by the dead-time (*t_d_*) of the detection system [10]. In this case, the exposure time required to reach the target SNR increases: $T=F^{2}SNR_{0}^{2}k_{0}^{-1}\left(1+k_{0}t_{d}\right).$ At the net of optical losses, the maximum theoretical count rate achievable depends on the number of fluorophores (*n*) and their lifetime (*k_sat_* = *nτ*^−1^). Using the limiting value for *F* = *1* and *k_sat_*, we can infer that the maximum acquisition rate for an ideal system to achieve a target SNR is $T_{\text{max}}^{-1}=SNR_{0}^{-2}n\tau^{-1}.$ We can introduce a second figure-of-merit for the relative acquisition throughput of a technique as the ratio between the theoretical acquisition rate for TCSPC and $T_{\text{max}}^{-1}$:

(3) $$A=F^{-2}\frac{\tau }{n}\frac{k_{0}}{1+k_{0}t_{d}}$$

The factor *F*^−*2*^ relates to the photon-efficiency of the system and it implies that when *F* > 1, a system requires *F*^−2^ longer acquisition times. The factor *nτ*^−1^ is the maximum achievable count-rate and the third factor is the effective count-rate achievable in the presence of pulse pile-up. There are two restrictions imposed by technologies on the magnitude of *k*_0_. First, some detection technologies cannot detect more than one photon per excitation pulse. Therefore, the highest average *k*_0_. will be limited to at least one or two order of magnitudes of the laser repetition rate to minimize the probability that two events occurs between two excitation pulses. For example, a typical Ti:Sapphire based system exhibiting a 80 MHz repetition rate might require limiting *k*_0_ to 800 kHz count rate. The effective dead-time for a FLIM system is a complex function of various parameters that also depends on the measured fluorescence lifetime [41]. Therefore, equation (3) is an approximation for the effects of pulse pile-up. Pile-up deforms the probability density function of the detected fluorescence emission unevenly because most photons are emitted immediately after the excitation pulse [42]. Although corrections for pulse pile-up have been reported (e.g., [8, 41]), it is often preferable to impose a limit in the acceptable pile-up losses, for example Δ < 0.05 [10], where Δ = *k*_0_ *t_d_* (1 + *k*_0_ *t_d_*)^−1^. Low Δ-values are preferable to avoid deterioration of lifetime accuracy (see also section 5). Low photon losses are also required to maintain high precision at lower excitation rates, thus limiting photobleaching and phototoxicity. The analytical description of *A* can be thus re-written as:

(4) $$A=F^{-2}\frac{\tau }{n}\frac{\Delta }{t_{d}}$$

**Figure 2:**
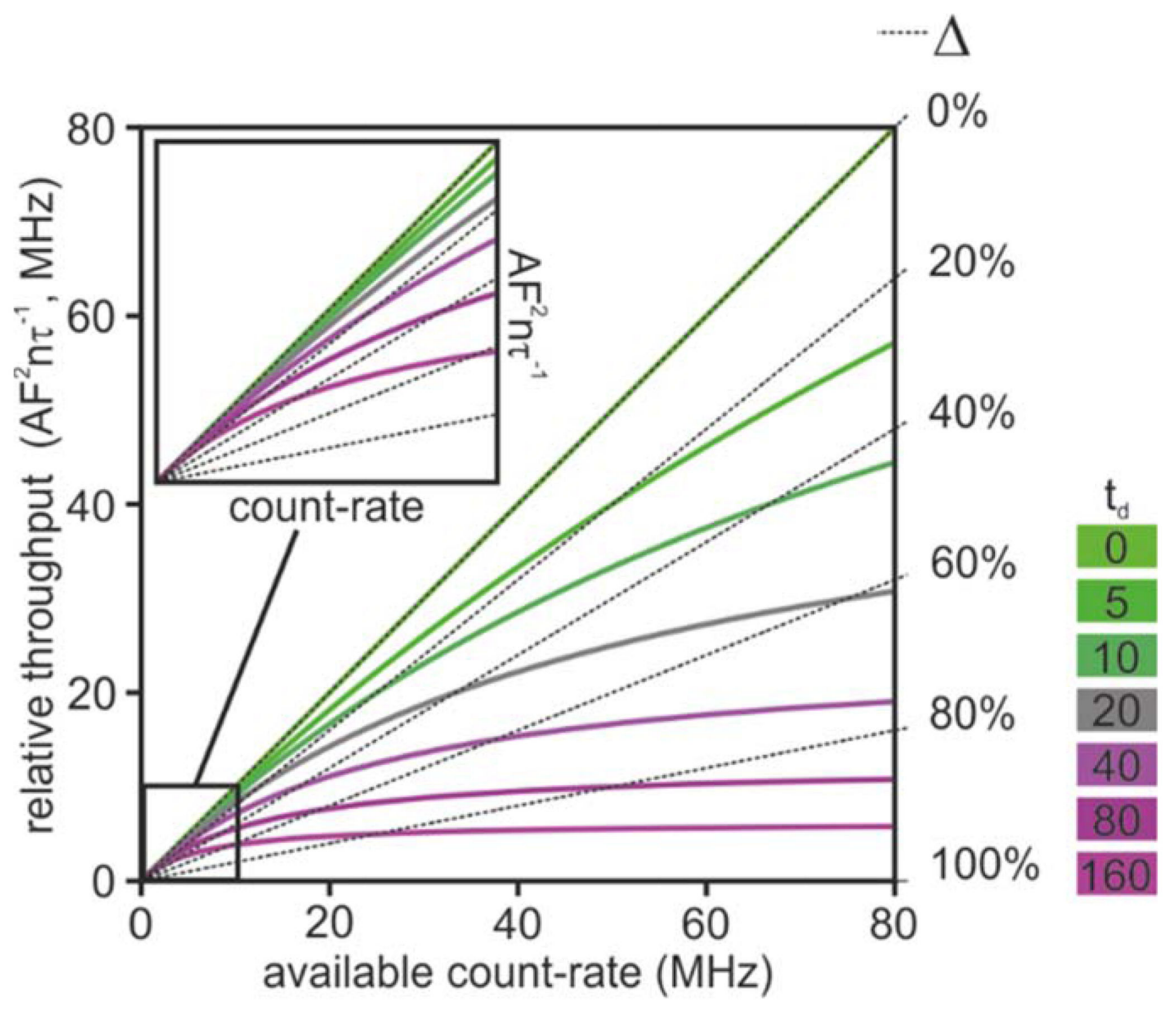
Acquisition throughput and single photon counting. The acquisition throughput is proportional to $∆t_{d}^{-1}$ and it depends on the photon rate impinging onto the detector. The solid curves depict the the effective count-rates at constant dead-time of the detection system from 0 ns (green) to 160 ns (magenta). The black dashed curves represent the effective count-rates given a tollerated loss of count-rate (from 0% to 100%). The inset shows the 10 MHz region of this plot.

This simple equation is rather instructive, as it exemplifies that with a finite dead-time, acquisition throughputs are rather limited in single photon counting. The highest acquisition throughput can be achieved with Δ = 1 (*i.e.*, the unrealistic case when the count-rate is so high that most photons are not detected), with an effective limiting count-rate imposed by the dead-time (*i*.*e*., $t_{d}^{-1}$). However, we have discussed how this can be deleterious and limited by accuracy and photodamage. At low values of *t_d_*, when Δ ~ *k_0_t_d_*, and with zero dead-time, *A* can thus achieve its maximum value. Figure 2 shows the losses of photons as a function of dead-time (coloured solid curves) or as a function of accepted losses (black dashed curves).

In summary, the acquisition throughput of a FLIM system operating in single phon counting mode is limited by the precision that a system can deliver (*F*^−2^), the accuracy (see section 5) and the maximum tolerated light dose (Δ) and the dead-time of the detection system (t_d_). Time-gating detection schemes can be quite efficient with low histogram resolution (4 to 8 time bins) of uneven bin-width [10, 39]. Usually, off-the-shelf electronics such as TDCs provides uniformly distributed historgramming capabilities; under these conditions, *F* converges to 1 on a sufficiently broad spectrum of lifetime values with several tens of bins [29]. For practical considerations (e.g., detection of raise, IRF, tail fitting) bin numbers around one hundred are advisable.

### 3.2 Imaging speed, dynamic range and resolution

The most evident advantage in increasing the effective count-rate in fast FLIM applications is the decreased acquisition time per image. However, this obvious advantage should not overshadow other, equally important consequences. FLIM microscopes based on point-scanning architectures are often limited to image single snapshots of relatively steady samples because of the comparatively long acquisition times required (~ one minute). The shorter acquisition times provided by fast FLIM electronics can be thus also invested in acquiring three-dimensional stacks or multiple fields of view (e.g., for high-resolution high-content imaging). Equations (3)–(4) describe the overall performance of a FLIM technique compared to the theoretical limit; the practical description of the speed (FR, frame rate) at which FLIM can acquire images is:

(5) $$FR=\frac{1}{N_{pixels}^{2}F^{2}SNR_{0}^{2}}\frac{\Delta }{t_{d}}$$

Equation (5) is evaluated at the net of time over-heads such as moving the stage of the microscope. Once imaging parameters such as the number of pixels within an image (here we consider a square image of *N_pixels_* per side), the desired target *SNR*, the efficiency of the technique (*F*) and the maximum permissible losses (Δ) are set, the acquisition speed will be inversely proportional to the dead-time of the single photon electronics and detector. A far less trivial consequence of the increased count-rate of a FLIM system exhibiting low dead-times is the gain of dynamic range. The dynamic range (*D*, measured in decibels) of single photon counting techniques is defined as the logarithm of the ratio between the detected count-rate (*k_detected_*) to the square root of the dark count-rate (DCR), *D* = 20 log_10_(*k_detected_ DCR*^−1/2^) [43]. DCR is the background count-rate generated by the detector in the absence of photons. With samples that vary considerably in intensity either across an image or over time, the speed of a typical FLIM is limited by the capability to image the brightest pixel without significant pulse pile-up. Therefore, high count-rate results in higher dynamic ranges and improved flexibility during imaging. To highlight the role of the instrument dead-time, we can define, *D_ref_* = 20 log_10_(Δ *DCR*^−1/2^) and the dynamic range can be thus expressed as a function of dead-time, once that Δ and DCR are fixed:

(6) $$D=D_{ref}-20\;{\text{log}}_{10}\left(t_{d}\right)$$

The smaller *t_d_*, the higher the dynamic range. We consider, for example, the use of a hybrid PMT that exhibits a DCR of ~600 Hz, an overload shutdown between 15–80 MHz, a dead-time <1 ns. By accepting pulse pile-up losses of 5%, *D_ref_* is equal to a negative contribution to dynamic range (around −54 dB) that grows in absolute value with increasing DCR and decreases when accepting higher losses. A typical maximum effective count-rate of TAC-based TCSPC with a dead-time of ~100 ns add 140 dB to the reference background, reaching D ~ 87 dB. Shorter dead-times of 10 ns and 1 ns result in significant gains in dynamic range reaching ~106 dB and ~126 dB, respectevly. In the same conditions, a SPAD detector with a DCR ~ 200 Hz and 10 ns dead-time, would deliver a dynamic range of about 91 dB and 111 dB, with electronics providing 100 ns and 10 ns dead-time, respectevely.

Furthermore, improved count-rates can lead also to better spatial resolutions because of the motion of biological samples. Slower FLIM systems might result in blurring, which is rarely quantified. The spatial resolution of a diffraction-limited confocal microscope is *R*_0_ = 0.6*λNA*^−1^ (different definition of resolution would not change the following discussion). For a green fluorophore (*λ* ~ 500 nm) and a high NA (~1.2), *R*_0_ reaches the typical values around 250 nm. In analogy to the dynamic range D, we can defein a figure-of-merit to describe the magnitude of image blurring as:

(7) $$B=20\;{\text{log}}_{10}\left(\frac{R}{b}\right)$$

The factor *b* is a characteristic distance describing sample motions. Biological samples move at very different time scales and with different types of motion. We consider Brownian motion as an illustrative case with *b*^2^ = 2D*_diff_* t representing the mean squared displacement. Structures like lysosomes and mitochondria exhibit mean squared displacements around 10^5^ nm^2^s^−1^ [44, 45]. As the time to reach a given SNR_0_ is $T=F^{2}SNR_{0}^{2}k_{0}^{-1}\left(1+k_{0}t_{d}\right):$

(8) $$B=B_{ref}-10\;{\text{log}}_{10}\left(t_{d}\right)$$

with

(9) $$B_{ref}=20{\text{log}}_{10}\left(\frac{R}{FSNR_{0}}\sqrt{\frac{\Delta }{2D_{\text{diff}}}}\right)$$

For a typical exposure time of one minute, *b* is ~3 *μ*m. For the shorter acquisition times that can be achieved with fast electronics (e.g., 6 s and 0.6 s), *b* is ~0.8 *μ*m and ~250 nm, respectively. Therefore, using equations (8), (9), we can estimate a loss of resolution equal to −20 dB, −10 dB or no loss, respectively. The differences in losses can be accounted for by the term −10 log_10_(*t_d_*) in equation (8), for instance by simply using detector dead-time electronic of 100 ns, 10 ns and 1 ns.

In summary, even when the raw speed (A) enabled by low dead-times of fast single photon counting systems is not required, lower dead-times enable microscopes to achieve a higher image throughput, dynamic range and to avoid resolution losses caused by blurring.

## 4 An open platform for fast scanning FLIM

### 4.1 Hardware

**Figure 3:**
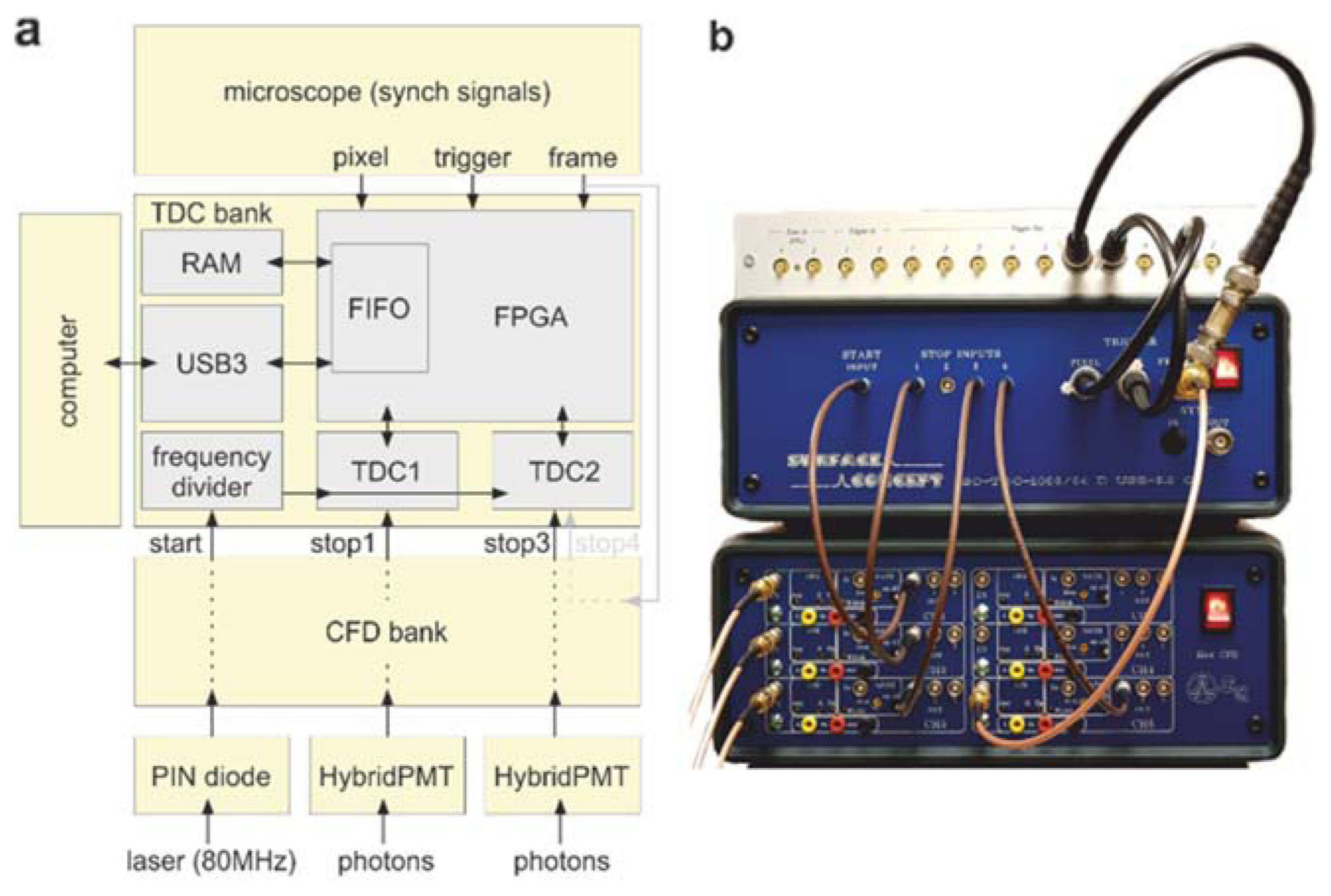
Electronics for fast biochemical imaging. (a) Diagrammatic representation showing the connections between the laser, the hybrid PMTs, the CFD bank, the TDC bank, the microscope and the computer. The internal representation of the TDC unit is adapted from Surface Concept drawings. (b) A photograph of the bench-top system with the CFDs (bottom unit), the TDCs (middle unit) and the microscope signal breakout unit (top unit). The three RF cables at the bottom left are connected to the PIN diode and the two hybrid-PMTs from top to bottom respectively.

We tested ELIS using a Leica SP5 multiphoton microscope equipped with two hybrid PMTs at the non-descanned port of the microscope, a Ti:Sapphire laser (Chameleon Vision 2, Coherent) and a Leica trigger signal breakout unit. This part of the system is commercially available and can be replaced by other commercial or open-hardware (e.g., [28]) solutions. We used a beam sampler (10B20NC.2, Newport), and a PIN photodiode (DET10A/M, Thorlabs) placed before the entrance of the laser scan-head to monitor the laser pulses which have been described in detail elsewhere [27, 46]. The electrical signals from the PIN diode and the PMTs are connected to a stand-alone six channel constant fraction discriminator (CFD) module by Surface Concept GmbH described in figure 3. The pre-conditioned signals are then fed to a bank of off-the-shelf multi-hit time-to-digital converters supplied by Surface Concept GmbH. Several systems are available from the manufacturer. We opted for a four channel TDC bank (SC-TDC-1000/04D), upgraded with an option to record pixel, line and frame trigger signals and USB3 for fast transfer of the time-stamps to the computer. The laser signal is connected to the ‘start’ input of the TDCs, while the PMT signals are connected to two of the TDC ‘stop’ inputs.

The pixel, line and frame trigger signals from the confocal microscopes are connected to the respective inputs of the TDCs. Furthermore, the frame trigger is also connected to a spare TDC ‘stop’ input. This channel of the TDC is used to correct for errors that we occasionally experience when generating image stacks from the stored photon streams.

The TDCs we selected for this open-hardware project are capable of 32 multi-hit operation for each of the two GPX chipsets integrated into the benchtop device. The nominal start-retrigger frequency is 9 MHz, which is insufficient for a laser repetition-rate of ~80 MHz. Therefore, a frequency divider embedded in the system can be configured to restart the TDC at a lower frequency than the repetition rate. We configured the frequency divider to 10 so that the TDCs are retriggered only at every eleventh laser pulse. The multi-hit TDC records a maximum of 32 stop events (*i.e.*, photons) for every 10 laser pulses, providing the capability to detect an average of 3.2 events per laser pulse. Therefore, each of the two GPX chipsets can detect unevenly distributed photon events, at an average maximum of 3.2 per pulse, relaxing the constraints of detecting less than one photon per pulse significantly. Furthermore, the equivalent to dead-time for a multi-hit TDC is the pulse-pair resolution. In our system, the pulse-pair resolution is 5.5 ns for each ‘stop’ input (or half of it, see section 5). While this is similar to the most recent high-end specialised solutions for FLIM, it is much shorter than the more traditional 80–100 ns values still broadly in use.

### 4.2 Software

**Figure 4:**
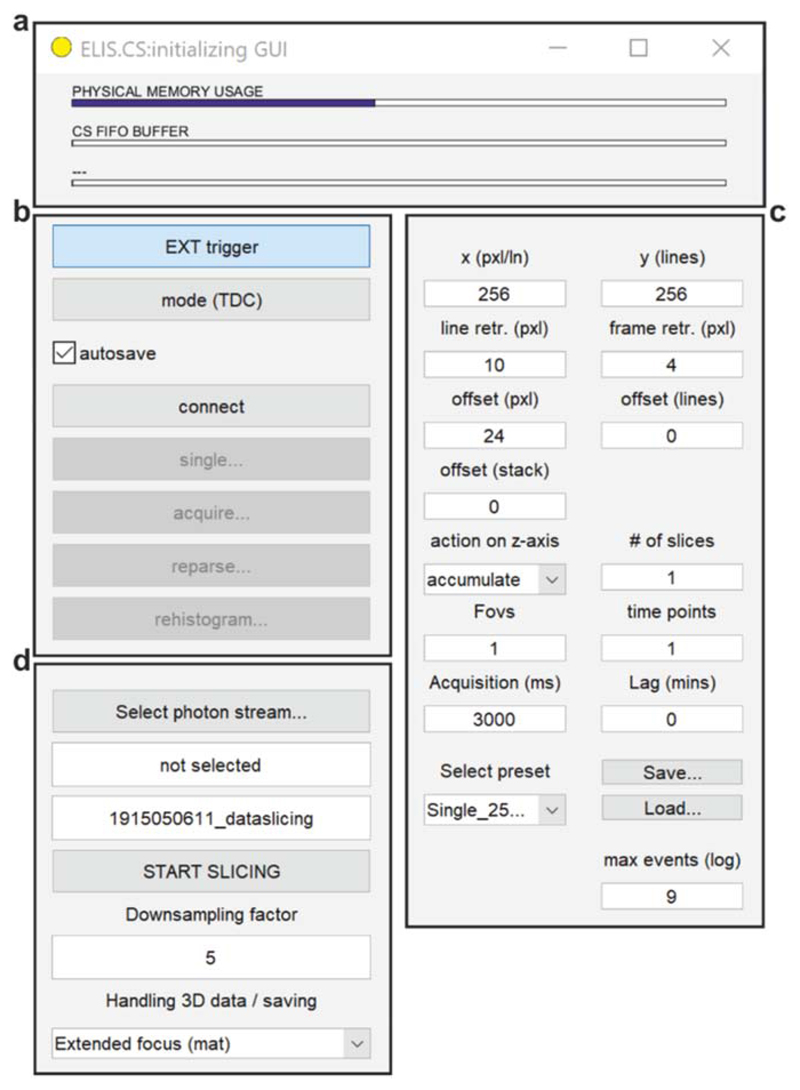
ELIS graphic user interface. (a) ELIS communication interface, (b) the main acquisition interface controls, (c) the imaging parameter interface and (d) ELIS.BOT described in the text.

ELIS (*ELISbeta1.1-build9* at the writing of this work) is a MATLAB (MathWorks) toolbox available at the GitHub repository ‘alesposito/ELIS’. ELIS has two main programs, ELIS.CS and ELIS.BOT. ELIS.CS (*elis*_*cs.m*) is the software that controls the hardware during acquisition and also permits the user to build FLIM images for individual single-frame acquisitions (figures 4(a)–(c)). ELIS.BOT (*elis*_*bot.m*) is the software that reconstructs images from buffers of photon streams for the more complex acquisition protocols that we often utilise, such as multi-colour, three-dimensional time-lapse acquisitions on multiple fields of view (figure 4(d)). When the control software is executed, several initialisation scripts are first called to load libraries for the hardware control and to set values of constants, error codes, and instrument parameters. Subsequently, ELIS.CS invokes a simple graphical user interface (GUI) to help the user execute a FLIM experiment. ELIS relies on a communication interface that reports on the system status, memory and FIFO buffer states, acquisition timing and analysis progress, depending on the context of operation (figure 4(a)). In the main acquisition GUI (figure 4(b)) the user can select betweend different modes of operating the TDCs.The hardware can start acquiring data upon an ‘external trigger’, *i.e.* when the laser scanning microscope starts collecting an image. The hardware can operate in a TDC mode, where photon- and trigger-events are streamed to the computer from a first-in-first-out buffer, or finally, in a FLIM mode where the histogramming of the images is performed in hardware. We have primarily developed the TDC modality aiming to support complex acquisitions on the laser scanning microscope. Images are then reconstructed in post-processing.

Once the operating modality is selected, the computer will establish a link with the TDC hardware. First, ELIS.CS compiles a configuration file (*elis.ini*) that is then transferred to the TDCs for the reconfiguration of its FPGA. Subsequently, a data pipeline is established where time-tagged events are continuously transferred from the TDC to an internal buffer as they occur and then streamed to the computer on demand. Data is then accumulated in the memory of the computer and histogrammed during post-processing (‘single’). The ‘reparse’ and ‘rehistrogram’ options aids the user to reconvert the buffer into parsed events and into images, respectively. For longer or more complex experiments, the data stream can be broken-up into smaller streams and stored on the hard-drive permitting continuous operation of the microscope (‘acquire’). In this case, the data stream is batch processed by ELIS.BOT which slices the stored buffer of photon events into individual image frames (when the ‘autosave’ option is active), each with in-pixel emission decays and up to three colours in its current implementation.

An imaging parameter interface (figure 4(c)) controls the imaging modality and histogramming of the datastreams by defining the number of pixels of the image, the pixel clock and frame clock counts lost during retracing. The offset values set the number of empty pixels, lines or frames to add to each acquisition to ensure an image is histogrammed with appropriate dimensions. Furthermore, the imaging parameter interface controls the histogramming on the z-axis (accumulate or generate z-stack), the number of z-sections, fields of view and time points. These parameters control the parsing of the data stack. The acquisition time and the lag time controls the timing of the TDC during acquisition, permitting ELIS to time out if there is an issue with acquisition and to wait in stand-by for a retrigger signal from the scan head (e.g., in a timelapse acquisition). The ‘max events’ input defines the length of the FIFO; tools to select preset values, saving and loading presets are also provided.

Finally, the GUI of ELIS.BOT permits the user to select a data stream, display the name of the current data stream, generate and display a randomly named folder to avoid overwriting data and handle 3D volumes either by accumulating it (extended focus) or generating 3D stacks downsampled by a given factor. ELIS.BOT also provides the user with a basic GUI (not shown) to display the progress and success of slicing individual frames over large datasets.

## 5 IRF and accuracy of fast FLIM

### 5.1 Instrument response function

**Figure 5:**
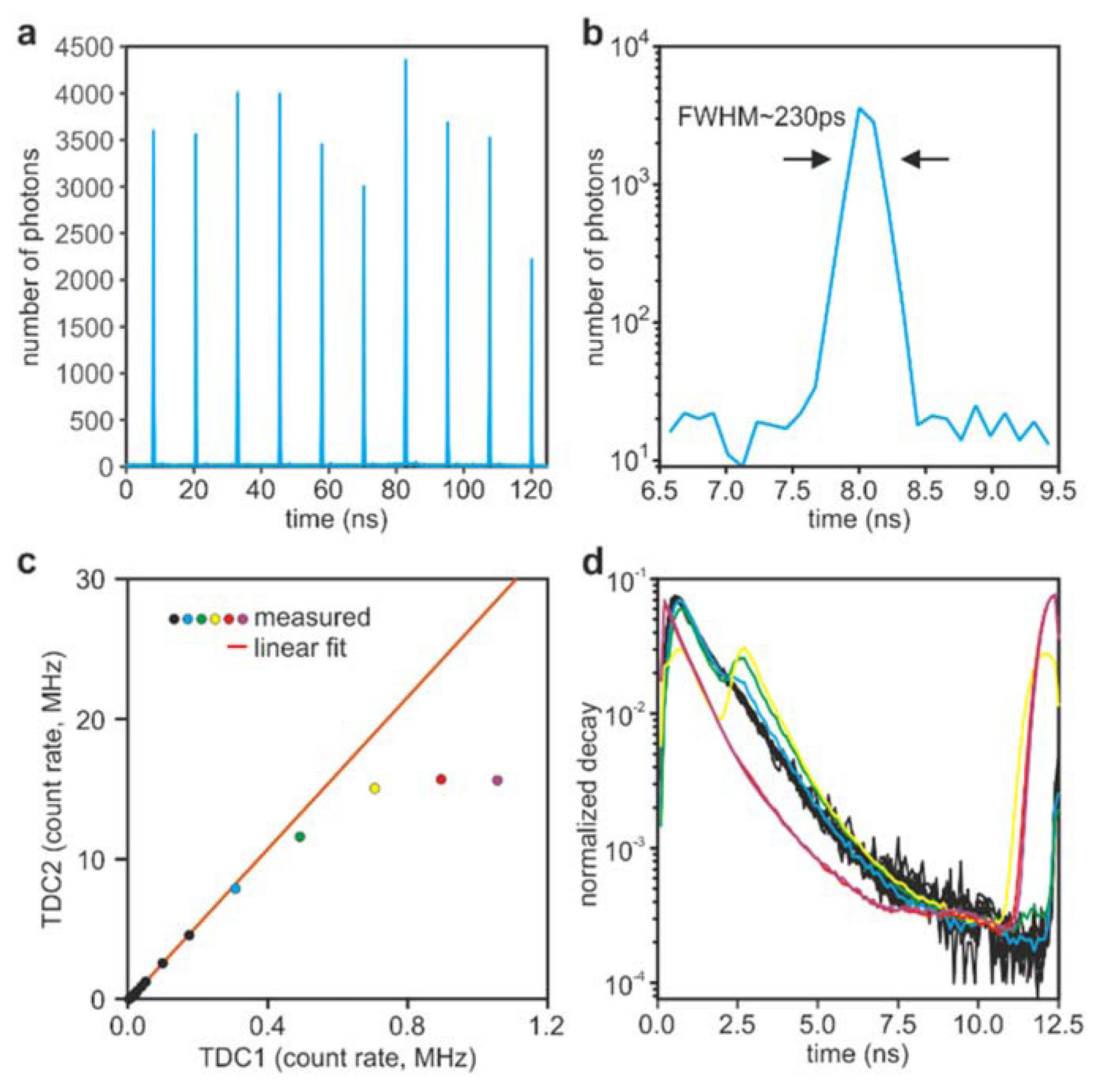
System performance. (a) IRF measured by aSHG signal scattered by KDP crystals. The ten peaks are a consequence of the frequency divider used to retrigger the TDCs. (b) The first IRF is shown zoomed-in in logarithmic scale. (c) Count rates detected by the first and second chipsets detecting a low (TDC1) and a high (TDC2) signals. The estimated count rates are marked with a solid circle, and experimental points exhibiting non-linearities are colour coded as a reference for panel (d). The solid red line is the linear fit computed on the first experimental points (black circles). (d) Fluorescence lifetime traces computed on the photon counts shown in (c), manifesting important non-linearities at high (>8 MHz) count rates.

The instrument response function (IRF) is the response of the electronics and detector to a (Dirac-like) pulse, and it is dominated by the transit time spread in the detector and the time jittering of the electronics. We have discussed how hybrid PMTs are the detector of choice for fast TCSPC. Hybrid PMTs exhibit an IRF with a full-width at half-maximum (FWHM) as low as a few tens of picoseconds. However, the hybrid PMTs of high quantum efficiency (>40%) in the visible range typically exhibit a transit time spread FWHM of ~120 ps [47, 48]. ELIS exhibits an IRF of about 230 ± 35 ps (figures 5(a), (b)).

This implementation of ELIS utilizes a hybrid PMT with a nominal IRF of 120 ps, we can thus extrapolate that the net contribution of the electronics of the system IRF is ~200 ps FWHM. At the time of the writing of this work, not all suppliers of fast FLIM electronics fully disclose specifications. However, the specifications for the PicoQuant GmbH MultiHarp 150, on which their RapidFLIM is based, is ~80 ps root-mean-square (RMS) of electronic noise, and the LaVision BioTec GmbH fast FLIM (FLIM X16) exhibit an FWHM of ~300 ps using a multi-anode detector. Considering that FWHM ~ 2.35 σ, that RMS and FWHM sum in quadrature, and that multi-anode arrays have a IRF of ~200 ps FWHM, we can infer that these low dead-time systems exhibit a broader IRF compared to top-in-class photon counting cards (even from the same manufacturers), at least within the limitations of current technologies. A broader IRF results in a worse limit of detection (the smallest measurable fluorescence lifetime increments) and in accuracy losses when not properly compensated [41]. However, many biomedical applications rely on the measurement of nanosecond-lived excited states of fluorophores, which is far from the limit of detection imposed by a ~200 ps IRF.

### 5.2 Count-rates

**Figure 6:**
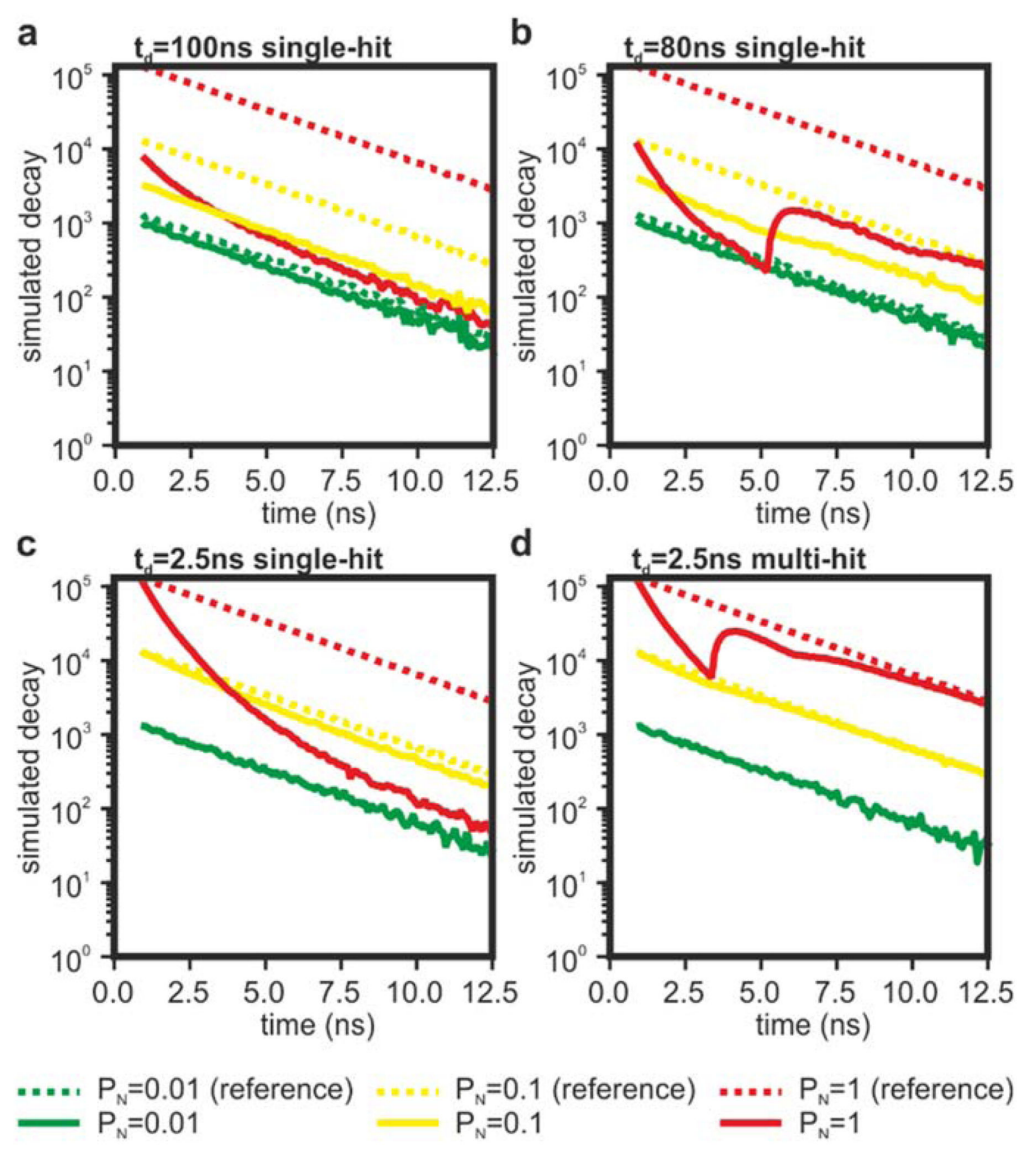
Montecarlo simulations of photon-losses and decay distortions. Simulated decays of a 3 ns fluorophore measured with electronics dead-time of 100 ns (a), 80 ns (b) 2.5 ns (c) and 2.5 ns with multi-hit capabilities (d). Dashed and solid traces are reference decays computed in the absence or the presence of dead-time, respectively. The results of simulations with low, high and very high count-rates are shown in green, yellow and red, respectively.

**Figure 7:**
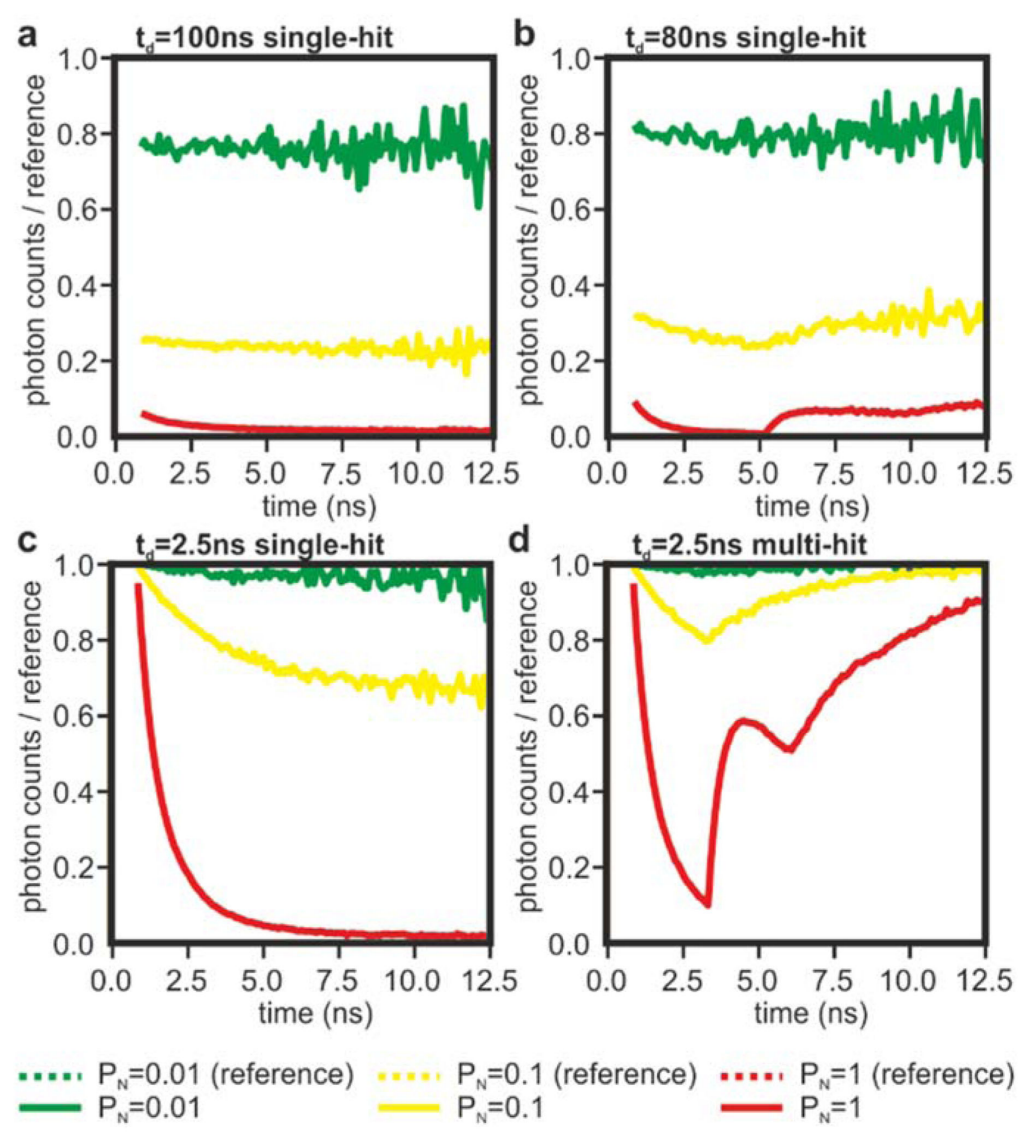
Monte Carlo simulations and normalized decays. Simulated decays of a 3 ns fluorophore (see figure 6) measured with electronic dead-times of 100 ns (a), 80 ns (b) 2.5 ns (c) and 2.5 ns with multi-hit capabilities (d). The ratios of the distorted to the reference traces (figure 6) with low, high and very high count-rates are shown in green, yellow and red, respectively.

With a nominal pulse-pair resolution of 5.5 ns, the time-to-digital converters utilised in this work are capable of reaching a burst count-rate of 180 MHz. However, the specifications of the TDCs state a maximum count-rate of 15 MHz per channel, limited by data transfer. We tested this capability by imaging a blue acrylic plastic slide due to its high brightness and resistance to photobleaching. We split the fluorescence intensities onto two detection channels with the use of a dichroic mirror (cut-off wavelength at 505 nm), and two bandpass filters (483/32 nm and 535/30 nm). On average, the blue-shifted channel detected ~ 5% of the total signal, and it was used as a low count-rate reference signal (TDC1 in figure 5(c)). The photon counts that is detected simultaneously by the second channel (TDC2) are plotted against those from TDC1 at varying laser powers and shown in figure 5(c). As expected, the count-rate saturates at around 15 MHz, with significant deviations from linearity visible from around 8 MHz. While the data transfer to a computer is a clear limiting factor, loss of accuracy caused by pile-up is the dominant issue at high count-rates (figure 5(d)). At count-rates higher than 15 MHz (equivalent to 12.5% of the laser repetition rate in our system), we experience a large broadening of the IRF, thus imposing a limit of count-rate for our system at around 10 MHz, at least in its current implementation. However, at approximately 8 MHz, the traces exhibit a distortion at around 2 ns after the peak of the decay, which is about half the nominal pulse-pair resolution of the TDCs (see also [49]). Therefore, we attributed this effect to the effective dead-time of TDC electronics. To test this hypothesis, we ran Monte Carlo simulations modelling the operation of a TCSPC system with a dead-time of 100 ns (figures 6(a) and 7(a)), 80 ns (figures 6(b) and 7(b)), 2.5 ns (figures 6(c) and 7(c)), and 2.5 ns with multi-hit capabilities (figure 6(d) and 7(d)). Simulations were carried out with a probability of detecting one photon per laser pulse (P_N_) equal to 0.01, which is considered a safe mode operation of TCSPC to avoid pulse pile-up artefacts, in addition to the higher values of 0.1 and 1. Even at P_N_ ~ 0.01 (*i.e.* ~ 800 kHz in our example), the relatively high dead-times of traditional electronics (~80–100 ns) cause significant photon losses (~20%). However, at this safe level of count-rate, the decays do not show significant distortions. At the higher count-rates, the photon losses continue to increase and significant distortions become observable in the decay curves.

Notably, when the dead-time is a multiple of the period of the laser pulses, the photon losses increase monotonically along the decay (figure 6(a)) giving the appearance of faster decay times [50, 51]. Dead-times that are not multiples of the laser pulse period introduce transient distortions within the traces rather than a monotonic distortion (figure 6(b)) [49]. While these transient distortions are striking when compared to the expected decay curves, the appearance of these ‘wobbles’ may aid the user in identifying pulse pile-up in the measurement. Furthermore, from a qualitative perspective, traces containing ‘wobbles’ may improve the accuracy of lifetime values computed from fitting algorithms by being less biased at the tail end of the decay and better approximating the slope of the reference signal.

We also simulated TCSPC with a dead-time of 2.5 ns to evaluate the performance of ELIS and commercial systems with very short dead-times. When compared to systems with longer dead-times, a dead-time of 2.5 ns (figure 6(c)) significantly limits photon-losses, thus providing higher precision; however, as the dead-time of the electronics is less than the period of the laser pulses, the traces are significantly distorted at higher count rates and require computational corrections for accurate lifetime calculations [41]. When we add in the capability to record multiple photon events per laser pulse, both the acquired photon budget is increased and the losses in accuracy are ameliorated (figure 6(d)). Even at low count-rates, fast TCSPC recovers a significant portion of the photon budget (figure 7), while the capability to record multiple events per laser pulse ameliorates the distortions of traces that cause significant losses of accuracy at intermediate count-rates (~8 MHz in the provided examples). While beyond the scope of this manuscript, we speculate that the non-monotonic nature of the distortions and the vast increase in photon budget (larger than 10-fold) might make the correction of fluorescence decay analyses more efficient and robust, even at high count rates (e.g., at 80 MHz for this example, see figure 7(a) *versus* 7(d)).

## 6 Biochemical imaging

**Figure 8:**
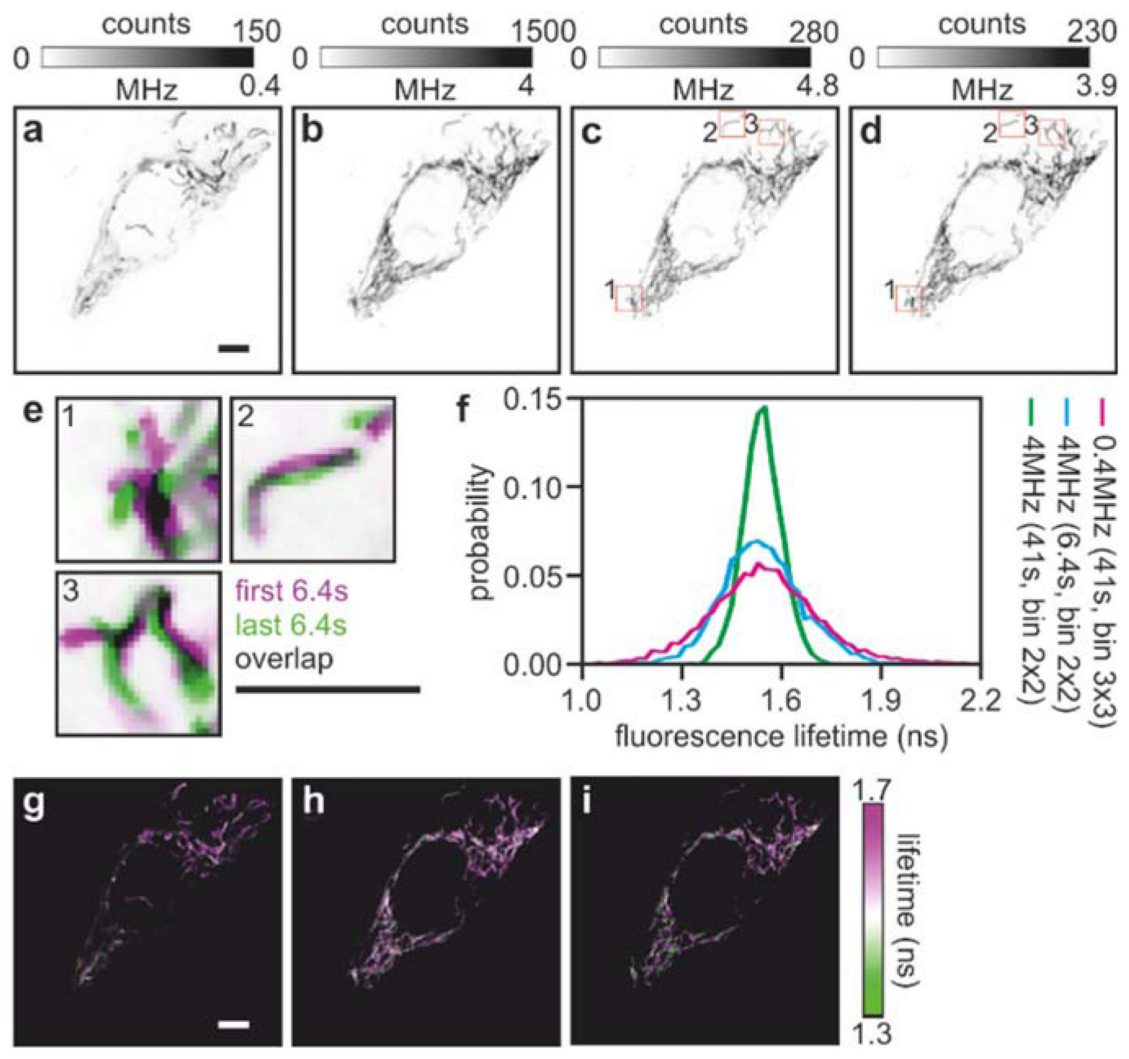
Fast FLIM imaging of dynamic processes. Intensity images of mitATeam transfected HeLa ells acquired at low (a) and high count-rates (b). The photon counts acquired during the first and last 6.4 s of the 41-second photon stream utilised for (b) are shown in (c) and (d), respectively. These two images are shown, overlaid in (e) and zoomed-in (3×) showing the dynamic nature of mitochondria. The fluorescence lifetime images of panels (a)–(c) are shown in panels (g)–(i) with their distributions shown in (f). Scalebar: 10 *μ*m.

To illustrate the possible advantages of fast TCSPC, we imaged HeLa cells expressing a genetically encoded sensor for adenosine triphosphate (ATP) that was targeted to the mitochondria (mitATeam) [30]. ATP is an essential metabolic product that stores chemical energy within the cell and is used for many biochemical reactions. Mitochondria, often refered to as the ‘power plants’ of the cell, are the main source of ATP production in mammalian cells. Mitochondria are very dynamic organelles, and mitochondrial dynamics and morphology affect cellular metabolism [52]. Cells expressing mitATeam permitted us to challenge the performance of our fast scanning FLIM system. Similar to other TCSPC systems, the laser scanning microscope repeatedly acquires images of the same field of view to accumulate a sufficient number of photons amenable to fluorescence lifetime analysis. Figure 8(a) and 8(b) show the photon counts images computed over 64 frames, accounting for 41 s of exposure time, with low (~0.4 MHz)) and high (~4 MHz) count-rates, respectively. From the data stream shown in figure 8(b), the integration of the first ten frames (figure 8(c)) and the last ten frames (figure 8(d)), each representing an acquisition time of 6.4 s, exemplify that within a fraction of time often used for typical TCSPC experiments, a sufficient number of photons can be collected with a Fast FLIM system for lifetime anlysis. Figure 8(e) shows an overlay of these two snapshots that are separated by 35 s, a time shorter than the usual acquisition time of a typical TCSPC image (1–2 min). The movement of mitochondria is apparent, illustrating that a faster acquisition time can reduce motion blur improving the spatiotemporal resolution in TCSPC. For illustration, the fluorescence lifetime images for figures 8(a)–(c) are shown in figures 8(g)–(i) and the fluorescence lifetime distributions are plotted in figure 8(f), showing how the capability to achieve higher count-rates can be used either to increase precision or acquisition speed in FLIM.

**Figure 9:**
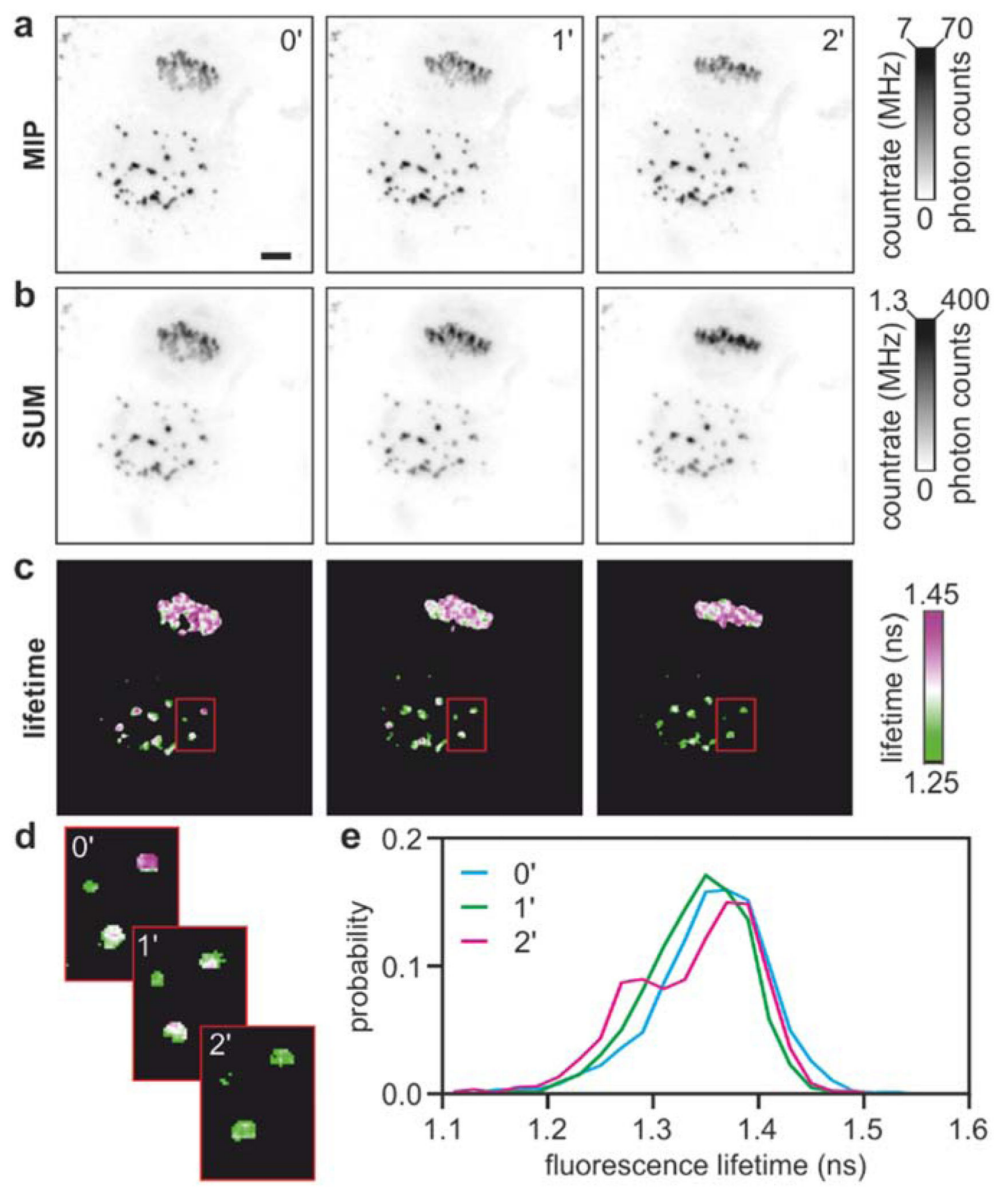
Fast FLIM and dynamic range. Maximum intensity projections (a) and total counts (b) and fluorescence lifetime (c) images of HeLa cells expressing a centromere-targeted Auorora Kinase B sensor. (d) 3×zoomed-in images corresponding to the red boxes in (c). (e) fluorescence lifetime histograms of the images shown in (c). Scalebar: 10 *μ*m.

To further exemplify how the capability to support high count-rates in single photon counting can be utilized, we also acquired images of HeLa cells expressing a sensor for the mitotic kinase Aurora Kinase B activity [32] targeted to the centromeres. During mitosis, cells segregate two complements of genomes into the two daughter cells by first pulling chromosomes at the centre of the cell (the metaphase plate) and subsequently separating the chromosomes into the two separating daughter cells. This is a highly dynamic process, regulated by several enzymes including mitotic kinases. In this case, we collected three-dimensional stacks made of 28 sections spanning 14 *μ*m within ~18 s; the acquisition was repeated three times at 0, 1 and 2 min. Figure 9(a) shows maximum intensity projections (MIP) for one cell undergoing mitosis adjacent to a cell in interphase.

Fast FLIM can capture this comparatively fast process, but – more importantly—it does so while providing a high dynamic range, with a maximum count-rate of ~7 MHz. Figure 9(b) shows the same data stacks but instead summed over the *z*-axis as ‘extended focus’ images. The maximum apparent count-rate in this case is about 1.3 MHz because some frames are dimmer than others. With samples that are sufficiently bright, the speed of single photon counting techniques is limited by the maximum count-rate acceptable within the brightest pixels, i.e. by the dynamic range of the photon counting technique.

Thus, with slower electronics, the acquisition speed of multi-dimensional datasets can be severely limited by the bright pixels within any area of interest of the entire dataset, including pixels that appear bright only in specific areas or times during a time-lapse experiment (e.g., cell shrinkage or chromatin condensation). Lifetime images of the data stack in figures 9(a), (b) are shown in figures 9(c), (d), and the histogram of each image is shown in figure 9(e). While differences in count-rates and brightness within these images are very pronounced, analysis of fluorescence lifetimes is still possible showing different activities for Aurora Kinase B in cells that overcame a mitotic block, cells that are still attempting to divide and between different centromeres over time. figures 8 and 9 illustrate that electronics with shorter dead-times can acquire FLIM images faster. More significantly, these examples illustrate how higher count-rates can be important not just to achieve faster acquisition speed, but to achieve a more balanced compromise between speed, precision, dynamic range and resolution, aspects that – in our opinion—are often neglected when discussing recent developments of fast FLIM techniques.

## 7 Conclusions

We have introduced a theoretical analysis to clarify and justify the need for faster single-photon counting electronics for FLIM applications. The advantage in acquisition speed of a single image is obvious but gains in precision, dynamic range and possible gains in the spatial resolution are equally important. These advantages also hold when limited photon budgets are available. Furthermore, we publicly share the hardware design and the software we have used in our laboratory for fast TCSPC. For several years, we have utilised this system not just for high frame-rate FLIM imaging, but primarily to benefit from better dynamic range in multi-colour time-lapse three-dimensional acquisitions and to minimise photon losses [27].

However, it is clear that the adoption of fast single-photon counting electronics requires some compromise, at least with the available technologies. First, it seems that fast single-photon counting technologies, to the best of our knowledge, results in a broader IRF compared to detectors and electronics optimised for small timing jitters. However, with a combined IRF of electronics and detector still in the ~200 ps range (FWHM), these FLIM systems can benefit the large majority of biomedical applications, except those that require the measurement of very short fluorescence lifetimes. The deterioration of the IRF, in any case, does not seem to be a limit imposed by the physics of single photon counting and, therefore, it is a limitation that might be overcome in the future (e.g., as shown with superconducting single photon detectors [53, 54]). A second compromise relates to accuracy. With the technologies characterized so far, single-photon counting electronics manifest distortions in the recorded traces resulting in loss of accuracy at very high count-rates. As for any other experimental technique, the experimenter (implicitly or explicitly) decides which level of accuracy and precision to accept. Fast single-photon counting instrumentation significantly limits photon losses, thus maximising precision. At low count-rates (P_N_ ~ 0.01), fast electronics seem to be mostly beneficial. At higher count-rates (P_N_ ~ 0.1), fast electronics deliver very high precision but with losses in accuracy that can be counteracted with multi-event capabilities. At the very high count-rates achievable with hybrid PMTs (P_N_ ~ 1), however, the loss of accuracy is significant. Unless electronics and detectors with even shorer dead times become available, this will remain a limiting factor in the adoption of fast scanning FLIM systems. It is also possible to ameliorate accuracy losses with appropriate modelling and data analysis as elegantly shown by Isbaner *et al* [41]. However, unless the causes of the problem are minimised (*i.e.*, pulse pile-up / dead-time) any strategy is likely to improve accuracy to the detriment of precision (e.g., fitting extra parameters or omitting photon counts likely to conceal pile-up).

Since fast FLIM technologies are becoming increasingly available commercially, we have chosen to highlight some of the possible shortcomings of this technology in this work. However, we should also clarify that this is a kind of ‘luxury problem’. While the ultimate performance at very high count-rates is yet to be confirmed with the development of new detection or analytical strategies, fast FLIM systems can deliver high precision, accuracy, dynamic range and spatio-temporal resolution in excess of typical TCSPC systems, seemingly only with a broadening of the IRF that might not be relevant to many biomedical applications. Different academic groups and manufacturers are exploring different strategies to speed-up quantitative biochemical imaging based on FLIM. These strategies include the multiplexing of time-to-analogue converters [55] (applicable also for TDCs), the parallelisation of laser scanning techniques (requiring lower count-rate to achieve fast imaging) [56–58] and wide-field FLIM technologies [21, 59, 60]. While each of the solutions described carry benefits and limitations, the analysis we have illustrated here applies to any of these approaches insofar as they are based on single photon counting. Ultimately, improving acquisition speeds while maintaining the robustness and quantification of FLIM for biochemical assays, it is not just important for several applications in the research laboratory but it is also fundamental for delivering improved assays for drug discovery [28, 61–63], healthcare applications [64–66] and similarly strategic applications. With this work, we aim to support the renaissance of time-resolved technologies that is currently fostered by the availability of better and faster commercial products and by upcoming new smart-pixel technologies, which we expect to impact applications in cell biochemistry and biomedical research in the near future.
